# COVID-19 compassion in self-isolating old age: looking forward from family to regional and global concerns

**DOI:** 10.1007/s42532-020-00053-4

**Published:** 2020-06-26

**Authors:** Ian Douglas

**Affiliations:** School of Environment, Education and Development, University of Manchester, 21 Taunton Road, Sale, Cheshire, M33 5DD UK

**Keywords:** Compassion, Family, Nurses, Multiple disasters, Emergency planning, Regional authorities

## Abstract

Self-isolating with my wife, I feel gratitude and compassion for all those supporting us, particularly those who regularly deliver our food and our immediate family members who check on us frequently. My compassion goes out to those on the “frontline”, particularly my niece and her daughter who are both nurses in a major hospital and who developed and recovered from COVID-19 symptoms. More broadly, I recognise that there are many communities that have had to cope with both geophysical and socio-politically created disasters while facing the COVID-19 pandemic, among then some young women bee-keepers in Uganda. In the UK context, I have great concern that severe funding cuts for regional and local public health services and disaster planning handicapped the country’s response to coronavirus and may have been a factor in the UK’s high coronavirus death rate. I see both positive and negative changes in air pollution and urban nature in our towns and cities, but also am concerned that we collectively may lose sight of the greater crises of climate change and species extinction. We have to work for a better future by taking forward the opportunities and lessons from our reactions to the pandemic. This leads to compassion for the yet unborn, our grandchildren’s children, who might enter a less habitable, more unequal less collaborative world than the imperfect one we now enjoy.

## Introduction

As a self-isolating octogenarian, my compassion goes to all the people who have enabled my wife and I to stay in our comfortable suburban home with its large 0.3 ha garden in Greater Manchester, UK, since 14 March 2020. I realise that we are far more fortunate than most people our age. In this essay, I consider my immediate personal reactions to the COVID-19 lockdown and the changes in the daily lives of my wife and I. I discuss how my family has been affected and how we now maintain contacts with our son and daughter. I then consider how the situation has revealed the consequences of the virtual elimination of any kind of public welfare and disaster preparedness at regional levels between central government and local authorities in England, UK. Drawing on my various past experiences (Douglas 1979, 2020; Douglas et al. 2010), I express concerns for vulnerable communities in multiple disaster situations and discuss how the changes happening in urban nature and the urban environment might be both beneficial and potentially problematic. I conclude by expressing the need to apply the concentration and coordination of the present campaign on COVI-19 to the potentially even more life-threatening climate emergency and associated extinction disaster.

## The personal situation and reaction

While I recognise that compassion pushes us to understand “how we have structured the world, and to ask how we can structure it better, not because we may suffer but because others are suffering and that is not how the world should be” (Galea 2020, p. 1), I begin with my personal situation. I feel somewhat guilty that because of my age and a recent illness, I am not able to do as much for others as I would wish. I share the Dalai Lama’s compassionate view that the *social* solution to the pandemic lies in our interdependence and the resulting happiness that comes from helping others (Seltzer 2020).

My immediate family members have seen that we have food and are well. More distant cousins have telephoned to check our welfare. Neighbours we had not previously met have asked if we need help. A wide variety of delivery drivers have brought milk, food, household goods, medicines, newspapers and books to the house: our days are marked by the particular items we expect to be delivered. Normally our son and daughter each bring food, and sometimes something special such as homemade cake or biscuits, that enliven our diet. There are telephone conversations with family and friends about the various delivery services for food. Instead of going to the shops, we have food from new sources: our milk delivery company which has interesting food from local suppliers; a major online grocery via our daughter; and from a local Italian restaurant chain. The geography of our retail experience has shifted. However, we feel that all of them are taking great care to deliver what we need in a safe and reliable manner.

### Novel ways of communicating

The internet has become an essential part of our health care and shopping. I use “AskmyGP”, a website by which I can send a message, sometimes with a photograph, to the local GP practice and receive an email, or a phone call, within a few hours. I had a specialist hospital appointment by telephone. I can order medicines online, and usually they are delivered by the pharmacy. All my contacts with these busy healthcare people have been excellent.

I have become used to Zoom meetings, both with the family and with the boards or members of organisations that I work with (the Commonwealth Human Ecology Council, Human Ecology Foundation, Manchester Geographical Society and UK Urban Ecology Forum). Two of the meetings involved members from Australia and New Zealand who seldom have the opportunity to express their views in person. For me, it is relief not to have to spend all day travelling to London and back for a meeting lasting 2 h or less.

Although my day-to-day life is relatively little changed by the pandemic, because my wife and I spend most of our days at home, we have had to cancel holidays and we miss having Sunday meals with our children and grandchildren and our occasional meals out at local restaurants. We are grateful to everyone involved in meeting the needs of people like us who are not leaving their homes.

### Implications for family members

I feel compassion for those on the “frontline” of health and social care. My brother has lived in Brussels, Belgium, since 1962. His elder daughter is a senior nurse in a major hospital there. Her own daughter is also working as a newly qualified nurse in the same hospital. Both of them came home from the hospital with a loss of their sense of smell and feeling tired. They had mild COVID-19 symptoms, from which thankfully, they both recovered and returned to work. My niece’s husband also became ill with COVID-19, but he too recovered. My niece had to have a further COVID-19 test but was able to continue working and in mid-May 2020 showed no COVID-19 symptoms. Another family member is caught up in plans to move house, having a place they want to move to, but being unable to progress the sale of their own house because of the social distancing problems.

## Emergency planning structures at the regional level

### Past austerity measures affecting disaster response

I also feel compassion for everyone who has been affected by the severe cuts in funding from central government that our local governments suffered (since 2010 in the UK), particularly those that have led to the diminution of local public health expertise. The losses extend to many metropolitan and regional strategic response and emergency planning bodies. In Greater Manchester, there used to be a strong Public Health team in each of the ten local councils and a fully staffed Greater Manchester Emergency Planning Unit (Table 1). The regional strategic health authorities that co-ordinated health services across local authorities and health authorities underwent many changes after 1974, finally being removed from the NHS system in 2013 (Table 2).

**Table 1:** Agencies responsible for emergency planning in Greater Manchester since 1974

Dates	Name and origin of organisation and relevant Act or Order	Territory of responsibility
1974–1986	Greater Manchester County Council	Greater Manchester
1986–1993	Emergency Planning Unit at Greater Manchester Fire and Civil Defence Authority	Greater Manchester
1993–2004	Six Districts of Greater Manchester had an agency agreement with the Fire and Emergency Planning Unit of AGMA (Association of Greater Manchester Authorities)	
2004 onwards	Greater Manchester Resilience Forum (Civil Contingencies Act, 2004)	Greater Manchester
2011 onwards	Civil Contingencies and Resilience Unit (CCRU) of AGMA	Greater Manchester
2014 onwards	All 10 Greater Manchester Districts make a political commitment to the Disaster Risk Reduction campaign of the United Nations International Strategy for Disaster Reduction (UNISDR)	Greater Manchester

**Table 2:** Changes over 75 years in the regional health strategic planning organisations for Greater Manchester

Dates	Name and origin of organisation and relevant Act or Order	Territory of responsibility
1947–1974	Manchester Regional Hospital Board (*National Health Service Act*, 1946)	Chester, parts of Lancashire and south Cumbria, NW Derbyshire, Greater Manchester
1974–1994	North-Western Regional Health Authority (*National Health Service Reorganisation Act* 1973)	Lancashire, Greater Manchester, Glossop
1994–2002	North West (Mersey and North West) Health Authority [*The National Health Service* (*Determination of Districts*) *Order* 1994]	Cheshire, Greater Manchester, Merseyside, south Cumbria and Glossop
2002–2006	Greater Manchester Strategic Health Authority (Health and Social Care Act 2001)	Greater Manchester and Glossop
2006–2013	NW England Strategic Health Authority [*The Health Authorities* (*Establishment and Abolition*) (*England*) *Amendment Order* 2004]	All of North West England
2013	Strategic Health Authorities abolished (*Health and Social Care Act* 2012)	No regional authority of any kind subsequently
2017	Conferral of certain public health functions of local authorities on the Greater Manchester Combined Authority (“the GMCA”). [*Greater Manchester Combined Authority* (*Public Health Functions*) *Order* 2017 (*The Public Health Order*)]	Move towards a metropolitan regional approach combining health and social care

Between 2013 and 2019 Public Health England, the body responsible for protecting the public from infectious diseases and environmental hazards, suffered a 40% cut in funding in real terms. The annual grant from the UK central government to local authorities for public health activities has been cut by 27.5% in real terms since 2015–2016 (Lawrence et al. 2020). Many considered that the combination of reorganisation and structural change has severely weakened England’s pandemic preparedness. I feel compassion for those who suffered from these impacts on the ability of the UK to react swiftly to declare a lockdown, get supplies into all appropriate settings and to be ready to assist more effectively in the sectors of cities that experienced public health workers knew to have high levels of ill health, inadequate housing, poverty and unemployment. For Greater Manchester, these issues are well understood (Robson 2016). The compassion, which leads us to say that the world is not how it should be, demands that we must find ways of helping people in such areas to avoid the higher national average infection rates they are experiencing in the COVID-19 pandemic.

### Organisational continuity

In Greater Manchester, for example, consistency in strategic planning coming from organisational continuity brought measurable benefits to local communities in such terms as responses to HIV/AIDS, diabetes and mental health in individual local authority district within the metropolitan area (Snow 2016). Many public health officers remained in post when the responsibilities for public health were moved from NHS local bodies to local municipal government authorities in 2012. Where this did not happen, constant reorganisation and shifts in policy meant that some local services deteriorated (Snow 2016). Nevertheless, the UK response to COVID-19 does not appear, in its initial stages at least, to have used local knowledge, to have identified particular pressure points and areas of high vulnerability. Despite reorganisation, local public health skills, understanding and experience in tracking and tracing infectious diseases could have been used.

Failure to secure, retain and use local knowledge is a major health and environmental issue. In urban environmental terms, from my experience with sewers, derelict land and waste disposal, the people who know where private and public sewers connected, where noxious chemicals had been stored and processed within a factory, and where waste materials, such as tar and contaminated rubble, had been dumped, are no longer in post and their knowledge is lost. I feel compassion for those who have been affected by delays and disruptions through lack of such local understanding and loss of long familiarity within and about the organisations, people and problems of particular regions and urban areas.

### City region disaster planning and response reorganisation

**Fig. 1:**
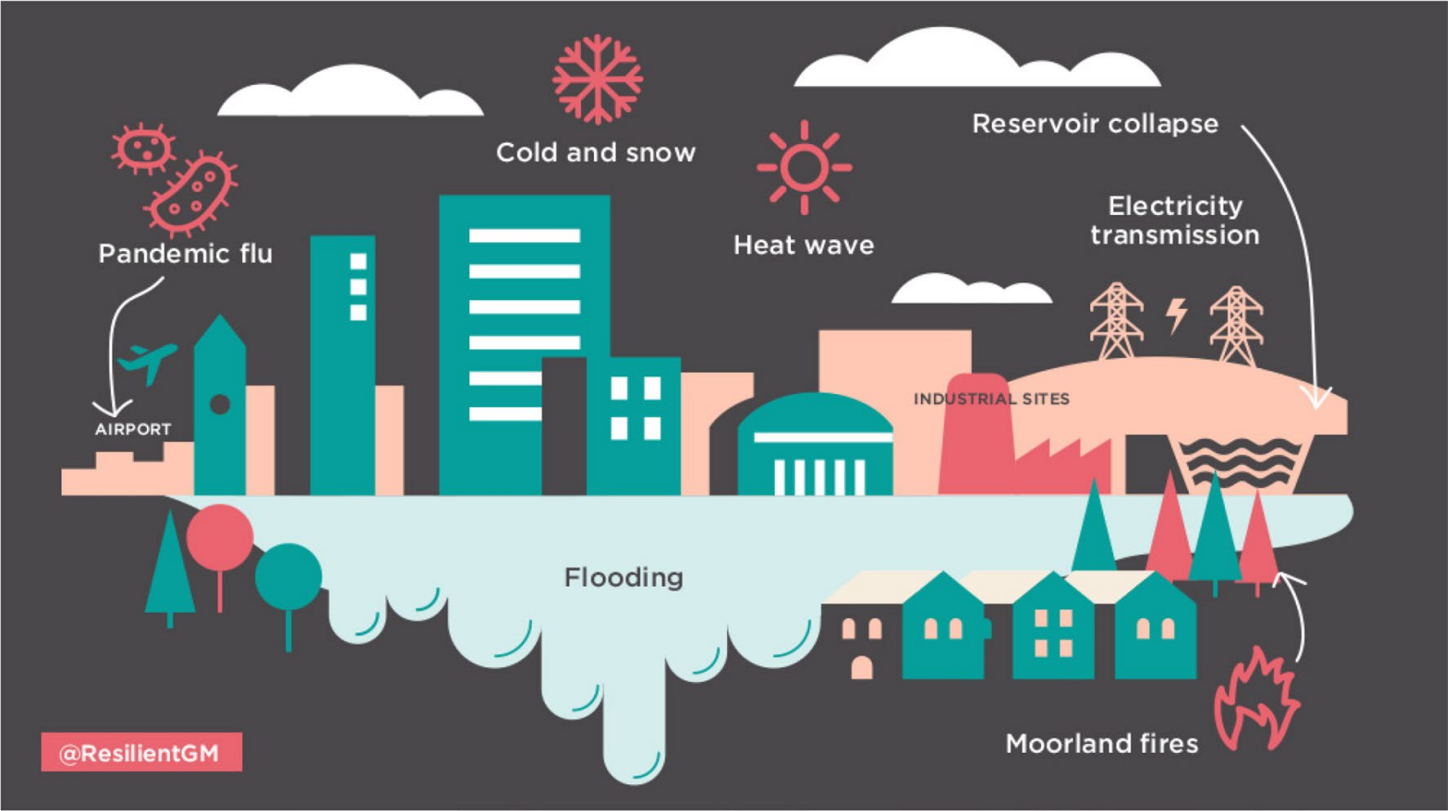
Emergencies, including pandemic flu, envisaged by the Greater Manchester Resilience Forum (https://www.100resilientcities.org/prepared-future-greater-manchesters-journey-emergency-preparedness-resilience/) (reproduced by permission of the Greater Manchester Combined Authority)

Regionalisation of emergency services co-ordination has its problems (Coaffee and Rogers 2008), but I see recent steps that seem to indicate that city region responsibilities may be redeveloping: emergency preparedness may be revived by the rolling programme of risk assessment set up by the Greater Manchester Local Resilience Forum after the 2004 UK Civil Contingencies Act (Oldham 2018). The Forum’s assessment includes preparations for pandemic influenza (Fig. 1) which may have come into play in the COVID-19 pandemic.

Big questions remain as to how well we learn lessons from past events. Awareness and concern about specific hazards decrease exponentially the longer the time span since an event has occurred. However, recommendations on the lessons that ought to have been learnt abound. The swine flu outbreak of 2009–2010 was declared a pandemic, but the 474 deaths across the UK that it caused fell far short of the worst-case scenario of 750,000 deaths. Some key issues emerging from the event (Boyd et al. 2012) were:

- The overly top-down national response;
- Importance of good coordination and collaboration between agencies at local level and between local organisations and regional bodies;
- Local flexibility to take account of local patterns of spread of infection;
- Clarity about which decisions will be taken locally, regionally and nationally;
- More use of social networking and digital technology to reach specific sections of the public.

## People facing multiple hazards

### Double disaster threat for a project in Uganda

I feel compassion for people working on projects in which the organisations I support are involved. In particular, I have endeavoured to support a pilot project which is training young unmarried mothers in bee-keeping in eastern Uganda. Since early March 2020, lockdown has prevented our local project supervisor from delivering the planned training sessions to the young women. Experts have not been able to visit the new hives to see how well they have been colonised by the bees. We have arranged some extra funding to support such visits as soon as travel from one village to another is permitted.

I was also concerned that there might be a double disaster facing the young women when Aljazeera reported that farmers in Uganda were facing a fresh onslaught of desert locusts in the first week of April 2020. In addition to the damage the insects might do, COVID-19 control measures mean that there were few staff available to implement field control measures. Fortunately, I was informed that the locusts had not invaded our project area. However, my anxiety continues as there is talk of a third generation of locusts in Uganda in June and July 2020 that would coincide with the start of the mid-year harvest.

### Cyclones, hurricanes and typhoons during the COVID-19 pandemic

“Normal” disasters are still occurring, but there is less space for them in the news media. Double disasters have happened in the western Atlantic, northern Indian and the south-western Pacific Oceans, where coastal communities already threatened by rising sea levels have been hit by tropical cyclones (hurricanes or typhoons). Many workers who had lost their jobs and who returned to their homes around the Bay of Bengal were hit by Cyclone Amphan on 19–21 May 2020, losing their homes, power and telecommunications connections, driving some families into destitution. On May 22 Hurricane Aaron hit the Bahamas, with winds of approximately 250 km h^−1^, killing 208 people and causing over $7.5 billion in damage. On May 24th Tropical Cyclone Mangga brought power outages and some damage to West Australian coastal communities from the Kimberley in the north to Perth.

In early April 2020, when many wealthy nations were individualistically in lockdown with closed borders, Tropical Cyclone Harold first damaged the Solomon Islands, then lashed the South Pacific island of Vanuatu, ripping off roofs and downing telecommunications, before moving towards Fiji and Tonga. The powerful cyclone made landfall in Sana province, an island north of Vanuatu’s capital Port Vila, with winds reaching up to 235 km an hour that flattened some buildings and left others roofless. This cyclone then levelled buildings and caused dangerous flooding across Fiji’s largest island of Viti Levu, before strengthening as it moved on to Tonga as a category 5 superstorm. Such tropical cyclones in the pacific can devastate the entire landscape of small islands.

When category 5 cyclone Harold struck Vanuatu in early April 2020, the country still had no confirmed cases of COVID-19. The islands had been closed to international travellers and had taken social distancing measures. In preparation for the pandemic, the UN had provided Vanuatu tents to treat patients and community outreach training. However, the stringent lockdown measures meant that the islanders had little outside help in dealing with the widespread devastation caused by the cyclone. Fortunately, their primary strategy to keep COVID-19 out largely worked. New Zealanders I collaborate with feel particular compassion for those in such situations for not only are they regularly hit by cyclones, but they are also already suffering by the rising sea levels and increased storminess caused by global heating and the consequent climate emergency. The islanders are not major contributors to the causes of climate change, but deserve our compassion because they are some of the most severely affected victims of its impacts.

## COVID-19 impacts of the urban environment and wildlife

### Urban air pollution and the global climatic effects of the pandemic

The impact of the COVID-19 lockdown on the climate is having both beneficial and harmful effects (Table 3). I am enjoying the clearer, bluer skies and the reduced air pollution resulting from decreased traffic and industrial emissions, but am concerned that national and international attention to the climate emergency has fallen. The COP 26 talks have been delayed until next year. Meanwhile, air pollution levels in China had rebounded to pre-pandemic levels by early June 2020 (Carrington 2020). The increasingly urgent need for a new international agreement on greenhouse gas emissions is being temporarily ignored, while the policing of illegal burning, waste disposal and other breaches of environmental legislation has been weakened.

**Table 3:** Advantages and disadvantages of COVID-19 lockdowns for urban ecology and humanity

Beneficial aspects	Source	Problematic possibilities	Source
Reductions in air pollution: NO_2_ levels in London’s ultra-low emission zone down by 40%	GU	Increase in volumes of waste collected leads to some councils incinerating more waste	GU
Carbon emissions in China down by 18% during lockdown, but rose again after end of March 2020	GU	In Wuhan, China, after restrictions were lifted in early April, pollution quickly returned to 2019 levels	g
Mumbai and Delhi experienced a reduction of 40–50% in nitrogen dioxide (NO_2_) compared to 2019	h	As economies eventually recover, there is likely to be an “emissions surge” which will leave the environment worse off	i
During the lockdown, aerosol levels were at a 20-year low in northern India	h	A risk that environmental policies will be relaxed during this time of crisis, as in the USA with President Trump relaxing emissions standards during the pandemic	i
In Israel: Nubian ibexes in Eilat city grazing in gardens; striped hyenas walking around Beersheeba; and wild boars with litters of piglets in Haifa	a	World’s wildlife will not be saved by economic downturn: need to ensure conservation moves to the top of the agenda in the post-pandemic world	c
Mountain lions in USA; wild boars in Italy; manatees in Costa Rica; a leatherback sea turtle, a jaguar and vulnerable great curassows in Mexico: all seen in cities and resort towns	b	Species associated with, and dependent on humans as a source of food, for example, urban gulls and pigeons may have fewer opportunities to forage on spilled foods and leftover takeaways	d
Fish-eating birds in Venice, Italy; wild boar in Bergamo, Italy; and of feral mountain goats in Llandudno, Wales	c	Monitoring work by British Trust for Ornithology staff and volunteers (and by many other organisations) has temporarily ceased during the lockdown	f
Reduced maintenance, such as urban road verge cutting and herbicide spraying, may allow wildflowers to proliferate providing additional food resources for pollinators	d	The surveillance and management of our precious wild places is considerably weakened	c
COVID-19 might lead to a reduction in the wildlife trade and the cleaning up of the wildlife markets, creating a win–win effect of both protecting species that are harvested from the wild and of reducing spread of new viruses	e	Community work has been severely reduced, and there is little formal consultation and capacity development in urban greenspace and wildlife management	j
Fewer wildlife deaths due to less traffic and more food available for garden wildlife (high demand for bird food from supermarkets)	l	Absence of volunteer activity means some urban natural areas are becoming undermanaged which could lead to scrub taking over sensitive areas of reed beds and grasslands and to invasive species such as Himalayan Balsam	k
Cleaner European air during Covid-19 lockdowns has helped to avoid 11,000 deaths	m	Major financial constraints on nature conservation bodies: decreased income, partly due to lower income for many households	l
Interventions to contain the COVID-19 outbreak led to air quality improvements that brought health benefits which outnumbered the confirmed deaths due to COVID-19 in China	n	More point-source pollution, especially where industrial companies close down, maintenance declines and inspections do not occur	l

a http://www.xinhuanet.com/english/2020-04/19/c_138990451.htm

b Diamant E, MacGregor-Fors I, Yeh P (2020) People Staying Home, Wildlife Occupying the Streets: Lessons from COVID-19 Lockdowns. *The Nature of Cities*, https://www.thenatureofcities.com/2020/04/22/people-staying-home-wildlife-occupying-the-streets-lessons-from-covid-19-lockdowns/ Accessed 10 May 2020

c Gardner C (2020) Nature’s comeback? No, the coronavirus pandemic threatens the world’s wildlife. The World Economic Forum COVID Action Platform https://www.weforum.org/agenda/2020/04/nature-s-comeback-no-the-coronavirus-pandemic-threatens-the-world-s-wildlife/ Accessed 10 May 2020

d Reid N (2020) How is coronavirus affecting animals? https://www.qub.ac.uk/Research/GRI/TheInstituteforGlobalFoodSecurity/institute-for-global-security-news/Howiscoronavirusaffectinganimals.html Accessed 08 May 2020

e Akpan N (2020) New coronavirus can spread between humans—but it started in a wildlife market. National Geographic, https://www.nationalgeographic.com/science/2020/01/new-coronavirus-spreading-between-humans-how-it-started/ Accessed 08 May 2020

f British Trust for Ornithology (2020) BTO and COVID-19. https://www.bto.org/community/news/202005-bto-and-covid-19 Accessed 08 May 2020

g Broom D (2020) Chart of the day: What happens to urban air quality when lockdowns lift? The World Economic Forum COVID Action Platform https://www.weforum.org/agenda/2020/05/coronaviris-covid-19-air-quality-cities-lockdown/ Accessed 07 May 2020

h First Post (2020) https://www.firstpost.com/tech/science/nasa-european-space-agency-data-show-drop-in-air-pollution-in-india-during-covid-19-lockdown-8304391.html Accessed 10 May 2020

i Barrett T (2020) Air quality making headlines during the coronavirus lockdown, https://airqualitynews.com/2020/05/04/air-quality-making-headlines-during-the-coronavirus-lockdown/ Accessed 12 May 2020

j Mark Champion, Wigan Projects Manager, Lancashire Wildlife Trust, UK (personal communication, May 2020)

k Richard Salisbury, Park Ranger, Manchester City Council, UK (personal communication, May 2020)

l Shirley P (2020) Is nature really gaining ground from the lockdown, or just being noticed more and getting temporary breathing space? ECOS 41(4) https://www.ecos.org.uk/liberating-nature-false-hopes-from-the-lockdown/ Accessed 08 May 2020

m Holder M (2020) Business Green, https://www.businessgreen.com/news/4014659/study-cleaner-european-air-covid-19-lockdowns-helped-avoid-deaths Accessed 06 May 2020

n Chen K, Wang M, Huang C, Kinney PL, Paul AT (2020) Air Pollution Reduction and Mortality Benefit during the COVID-19 Outbreak in China. medRxiv 2020.03.23.20039842; doi: 10.1101/2020.03.23.20039842

GU The Guardian (newspaper) April 2020

Back in the cities, there are conflicting stories about urban nature (Table 3). Those people at home lucky enough to have their own gardens or well-vegetated parks in which to walk have heard more bird song and seen animals wandering on to quieter streets and greater numbers. On the other hand, crucial work in removing noxious weeds, like Himalayan Balsam, may be greatly reduced in the UK where nearly 40,000 Wildlife Trust volunteers have to stay at home. Nature conservation organisations thus may not be able work on nature reserves, have little help with administration, and no outreach activities. If industrial firms close, or are forced to shut because of bankruptcies, maintenance of installations may cease and the risk of chemical and gas leaks will increase (Shirley 2020).

## How could our COVID-19 experiences help us change our behaviour to cope with the climate crisis?

The way governments have responded to the COVID-19 emergency shows that it is possible to change human behaviour. The amount of outdoor exercise now being taken by walking in the local area and using urban greenspaces gives me hope that in future people will recognise the health and psychological benefits of contact with nature in towns and cities, expressed in recent books (Clare 2020). However, I have compassion for those who write to newspapers urging acknowledgement of our collective failure to respond to climate change and identify its consequences. I also accept the need for all of us, at the personal, local, regional, national and global levels, to accelerate the mitigation and adaptation so desperately needed now to cope with the climate crisis that threatens a greater tragedy than coronavirus might have been.

We also need to build on the concerns shown among neighbours and the elements of national solidarity encouraged by politicians, to look beyond our own national concerns: the pandemic is a global phenomenon, just as the climate crisis and the Anthropocene mass extinction are (Scharf, 2020). I feel compassion for those who like me have witnessed enormous global changes in their own life times during which the pace of development, with its competition for the tallest, biggest and most profitable, has taken our eyes off the really important goal of sustaining a world that our grandchildren’s children will be able to enjoy. I have compassion for the yet unborn, who may suffer if we are not smart enough, not efficient enough, not caring enough, not serious enough about the ecosystems that support us, and all the biota with which we share the planet, to ensure this habitable world remains so for future generations. It may take a long time to “get back to normal”, but should we be trying to move on to a new situation of continuing compassion, care for others and care for our environment?
